# Comparison of various chemometric methods on visible and near-infrared spectral analysis for wood density prediction among different tree species and geographical origins

**DOI:** 10.3389/fpls.2023.1121287

**Published:** 2023-03-10

**Authors:** Ying Li, Brian K. Via, Feifei Han, Yaoxiang Li, Zhiyong Pei

**Affiliations:** ^1^ College of Energy and Transportation Engineering, Inner Mongolia Agricultural University, Hohhot, China; ^2^ Forest Products Development Center, School of Forestry and Wildlife Sciences, Auburn University, Auburn, AL, United States; ^3^ Laboratory Zhejiang Huadong Forestry Engineering Consulting and Design Corporation, Hangzhou, China; ^4^ College of Engineering and Technology, Northeast Forestry University, Harbin, China

**Keywords:** visible and near infrared spectroscopy, lifting wavelet transform, variable selection, response surface methodology, wood density

## Abstract

Visible and near-infrared (Vis-NIR) spectroscopy has been widely applied in many fields for the qualitative and quantitative analysis. Chemometric techniques including pre-processing, variable selection, and multivariate calibration models play an important role to better extract useful information from spectral data. In this study, a new de-noising method (lifting wavelet transform, LWT), four variable selection methods, as well as two non-linear machine learning models were simultaneously analyzed to compare the impact of chemometric approaches on wood density determination among various tree species and geographical locations. In addition, fruit fly optimization algorithm (FOA) and response surface methodology (RSM) were employed to optimize the parameters of generalized regression neural network (GRNN) and particle swarm optimization-support vector machine (PSO-SVM), respectively. As for various chemometric methods, the optimal chemometric method was different for the same tree species collected from different locations. FOA-GRNN model combined with LWT and CARS deliver the best performance for Chinese white poplar of Heilongjiang province. In contrast, PLS model showed a good performance for Chinese white poplar collected from Jilin province based on raw spectra. However, for other tree species, RSM-PSO-SVM models can improve the performance of wood density prediction compared to traditional linear and FOA-GRNN models. Especially for Acer mono Maxim, when compared to linear models, the coefficient of determination of prediction set ( $R_{p}^{2}$ ) and relative prediction deviation (RPD) were increased by 47.70% and 44.48%, respectively. And the dimensionality of Vis-NIR spectral data was decreased from 2048 to 20. Therefore, the appropriate chemometric technique should be selected before building calibration models.

## 1 Introduction

Visible and near-infrared (Vis-NIR) spectroscopy, which contains the visible and NIR regions, has been widely applied in agriculture, petroleum, pharmaceuticals, and life sciences, such as soil particle size determination (Gozukara et al., 2022), propane content prediction of liquefied petroleum gas (Dantas et al., 2013), polymorphic forms of fluconazole identification (Mansouri et al., 2021), plant stress detection (Liang et al., 2018), and examination of Zika virus (Fernandes et al., 2018). The visible region (380-750 nm) contains the information of the pigments such as anthocyanin and chlorophyll based on their specific absorption bands (Zahir et al., 2022). Meanwhile, the near-infrared light records the vibration of the hydrogen bonds, for instance, C-H, N-H, and O-H, which are the main components of samples. Therefore, when the Vis-NIR light strikes samples, the light absorbed by samples includes the information of pigments and hydrogen bonds, which can be used to predict samples’ components.

Wood density is an essential indicator for the assessment of wood qualities due to the relationship between the mechanical, optical, and chemical properties (Resquin et al., 2019). The traditional measurement of wood density is laboratory test based on the density formula (ρ=m/v, where ρ is wood density, m and v are the mass and volume of wood samples, respectively), such as China National Standards (GB/T 1933-2009), which is challenging because they require burdensome sample processings, meaning that it is a destructive behavior and difficult to measure numerous samples in a short time. In addition, wood properties are influenced by the tree species and geographical origins (climate, moisture, soil, etc.). Even within the same tree, there exist differences between juvenile and mature wood for density (Krajnc et al., 2021). These differences in wood properties have effect on end use and economic benefits. For example, the yellow rosewood (the raw material of classical Chinese furniture) grown in Hainan province is expensive than other locations due to its great quality (Huang et al., 2018). Therefore, it is necessary to analyze wood properties among different geographical locations and tree species, especially for native tree species and the Convention on International Trade in Endangered Species (CITES) listed species.

Many studies (Zhao et al., 2010; Zhao et al., 2012; Tigabu et al., 2020; Toscano et al., 2022) have demonstrated that Vis-NIR or NIR spectroscopy can be used to determine physical and chemical compositions, mechanical properties, wood microstructure, and seed quality over the years with the advantages of rapid, simple, and non-destructive detection for numerous samples. For the prediction of wood density using spectroscopy, various wood samples and chemometric techniques or the combination of these two sections are the main research directions. In terms of wood science, Schimleck et al. (2003) estimated air-dry density of green *Pinus taeda* radial samples with NIR spectroscopy, the coefficient of determination (R^2^) are 0.85 and 0.87 for green and dry wood, respectively. Additionally, in another study, Schimleck et al. (2018) found that air-dry density of *Pinus taeda* L increased from pith to bark at all heights based on NIR spectroscopy technology. As for chemometric techniques, Zhang et al. (2022) proposed a deep transfer learning hybrid method with automatic calibration capability (Resnet1D-SVR-TrAdaBoost.R2) to predict larch wood density in different moisture contents. Fernandes et al. (2013) compared the effect of two calibration methods (neural networks and partial least squares) on Pinus pinea density, the results demonstrated that neural networks was better than PLS technique. In addition, considering spectra quality and model accuracy, Li et al. (2022) analyzed various spectral pre-processing and multivariate calibration methods in the prediction of Chinese White Poplar density, the results showed that the best prediction was obtained by GRNN models combined with LWT and CARS method. These studies displayed that chemometric techniques are essential for NIR spectra analysis to better explore the relationship between spectra and properties.

The original spectra contain irrelevant information due to the interference of background and environment, therefore, chemometric methods are needed for Vis-NIR spectral analysis. The essence of Vis-NIR spectral data analysis is to extract useful information of components using the appropriate chemometric methods, which include pre-processing, feature variable selection, and multivariate calibration models. Pre-processing is an essential step for improving model prediction accuracy through converting raw spectral data into a new data set without interferences (Bian et al., 2020). The common used pre-processing techniques are multiplicative scatter correction (MSC), the first derivative, Savitzky-Golay (SG) filtering, detrending (DT), wavelet transform (WT) and standard normal variate (SNV) (Dotto et al., 2018; Li et al., 2020; Bian et al., 2022; Carvalho et al., 2022; Ling et al., 2022). Different results will be obtained using various pre-processing methods or their combinations due to the different mechanism and functions, thus, it is important to select the most useful method and prevent the phenomena of over-fitting.

A Vis-NIR spectrum of a sample with the region from 350 to 2500 nm includes 2151 spectral variables, the high-dimensional spectral data results in the “curse of dimensionality”. Therefore, feature wavelengths selection techniques should be employed to address the problem. Uninformative variable elimination (UVE), competitive adaptive reweighted sampling (CARS), genetic algorithm (GA), iteratively retains informative variables (IRIV), and successive projections algorithm (SPA) are among the feature variable selection methods that have been analyzed in recent studies (Centner et al., 1996; Araújo et al., 2001; Ielpo et al., 2017). In addition to these mentioned techniques, a hybrid method that combing two or more methods were made to simplify high-dimensional spectral data. For example, Yun et al. proposed a hybrid strategy based on variable combination population analysis (VCPA), IRIV, and GA to optimize key variables (Yun et al., 2015; Yun et al., 2019).

Multivariate calibration models are employed to analyze the relationship between the selected feature wavelengths and targeted properties. Generally, modeling approaches are divided into linear and non-linear models such as partial least-squares (PLS), principal component regression (PCR), artificial neural network (ANN), and support vector machine (SVM). Regarding the performance of linear and non-linear models, there may exist differences in prediction accuracy for the same sample because of the different strategies. According to the Beer-Lambert’s Law (A = ϵ× *l* × *c*, where A is absorbance,ϵ, *l*, and *c* are the molar absorption coefficient, path length, and concentration, respectively), there is a linear relationship between absorbance spectra and concentrations (Wang et al., 2011). However, the relationship between spectra and properties are complex or non-linearity due to the interference of environment, such as light scattering (Geladi et al., 1985). As for NIR spectra analysis, a linear method of PLS is usually used to model. If the residuals of the predicted model are normally distributed around zero then the PLS model is accurate. If the residuals will not be equally distributed around zero but will follow perhaps a”banana shaped” curve around zero or some unbalanced pattern, another calibration method or non-linear model will be employed to analyze the relationship between spectral data and properties. It is worth mentioning that non-linear calibration models have ability to perform linear analysis. What’s more, non-linear relationships between spectra and concentrations can be handled by the linear calibration models, but at the cost of the increasing of the multivariate complexity. However, the linear models are not always effective in the spectral data analysis when the noises are multiplicative. Additionally, spectral differences as different unknown samples or growing environment are complex. In this case, it is not enough to deliver the optimal solution only using linear techniques for such complex problem.

Recently, some new algorithms, such as random forest (RF) (Marta et al., 2022) and fruit fly optimization algorithm (FOA) (Li et al., 2019), were applied in the analysis of spectral data. Modeling methods are the same as pre-processing and feature variables selection techniques, they can be combined by two or three approaches to obtain the most suitable model. The limitations of modeling methods (linear and non-linear models) are the optimization of modeling parameters. For instance, the principal component number (PC_S_) and the selection of radial basis function (RBF) are the key step of PLS and SVM models, respectively.

In this study, various chemometric methods including a new spectral data de-noising (Lifting wavelet transform, LWT), four feature variables selection techniques (SPA, UVE, IRIV, and CARS), and two hybrid multivariate calibration models, i.e., generalized regression neural network (GRNN) optimized by FOA (GRNN-FOA), and PSO-SVM model optimized by response surface methodology (RSM-PSO-SVM), were compared simultaneously to obtain the most suitable chemometric technique for wood density prediction among different tree species. In addition to, the effect of geographical location on wood Vis-NIR spectra was investigated to predict wood density based on Vis-NIR spectroscopy.

## 2 Materials and methods

### 2.1 Study area and wood sampling

**Figure 1:**
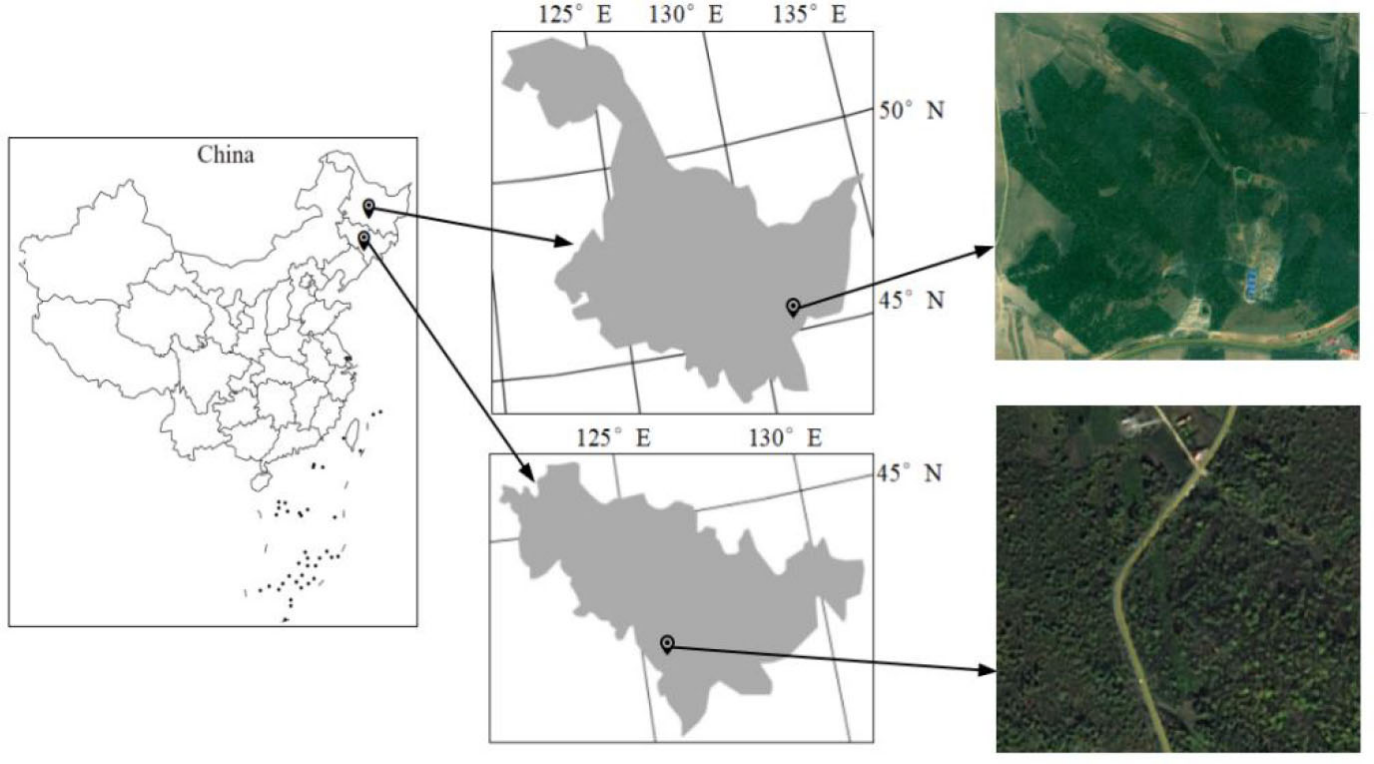
Geographical location of wood samples.

A total of 37 trees were collected from two physiographic regions of China (**Figure 1**). The main tree species comprising Populus davidiana, Ulmus pumila L., Acer mono Maxim., and Tilia tuan Szyszyl. were obtained from Jilin province. Populus davidiana and Larixgmelinii were simultaneously made from Heilongjiang province to analyze the influence of locations on wood density determination. The study area have east Asian monsoon climate and temperate continental monsoon climate, respectively. Wood disks with five-centimeter-thick were made from the stump up to the top at 1 m intervals. In total, 530 wood samples with the dimensions of 2×2×2 cm^3^ (tangential, radial, and longitudinal) were generated and then air-dried in laboratory for three months.

### 2.2 Vis-NIR spectra collection and wood density measurement

The reflectance spectra of wood were measured from the cross-section using a portable spectrometer (LabSpec, Analytical Spectral Devices, Inc., Boulder, USA). The wavelength range and spectral resolution are 350-2500 nm and 3 nm @700 nm, 10 nm @1400/2100 nm, respectively. A white panel was used for instrumental calibration every fifteen min. Three random spectra were collected from each sample and the average spectrum was regarded as the raw spectrum. Wood density was measured according to China National Standards (GB/T 1933-2009).

### 2.3 Chemometric techniques

#### 2.3.1 Spectral pre-processing

Wavelet transform (WT) is a powerful signal analysis technique in data compression and spectral de-noising (Mojsilovic et al., 2000). Lifting wavelet transform (LWT), the second-generation wavelet, can conquer the weakness of traditional WT. The inconspicuous information are magnified by LWT with the advantages of high computation speed and small memory. Wavelet function, wavelet order, and decomposition level (k) are essential for spectral de-noising using LWT and WT. Wood spectra with the bands ranging from 350 to 2397 nm (No. of wavelengths = 2048) were used to analyze in this study. Assuming that decomposition level and wavelet order are 8 and 3, respectively, four wavelet function including Haar, sym3, bior1.3 (biorNrNd, Nr: the wavelet order in decomposition process, Nd: the wavelet order of reconstruction wavelet), and db3 were compared in this study. The optimal wavelet function was determined by the performance of partial least squares (PLS) models. Then the optimal wavelet order and decomposition level were obtained based on the best wavelet function.

In order to better analyze the suitability of LWT, wood spectra were processed by three traditional pre-processing methods including WT, SNV, and MSC. The main parameters of WT were the same as LWT. LWT and WT were implemented in Matlab R2010b (MathWorks, Natick, USA). MSC, SNV, and PLS were performed with Unscrambler V10.4 (CAMO Software AS, Oslo, Norway).

#### 2.3.2 Feature variables selection

After the optimal de-noising technique was determined, four regular variable selection algorithms (i.e., UVE, CARS, IRIV, and SPA) were used to select the feature wavelengths of wood density. The selection strategy of UVE and CARS are filter-based and MPA-based, respectively. SPA is based on extreme value search and forward selection. In contrast with SPA, backward selection and MPA-based are employed by IRIV (Yun et al., 2014). These four variables selection methods were implemented with Matlab R2010b. The performance of these methods was analyzed by the PLS models according to the values of the coefficient of determination (R^2^), root mean square error (RMSE), relative prediction deviation (RPD), and relative standard deviation (RSD). Generally, a higher R^2^, RPD and lower RMSE, RSD value indicates a good predictive ability (Yan et al., 2013).

#### 2.3.3 Machine learning models

**Figure 2:**
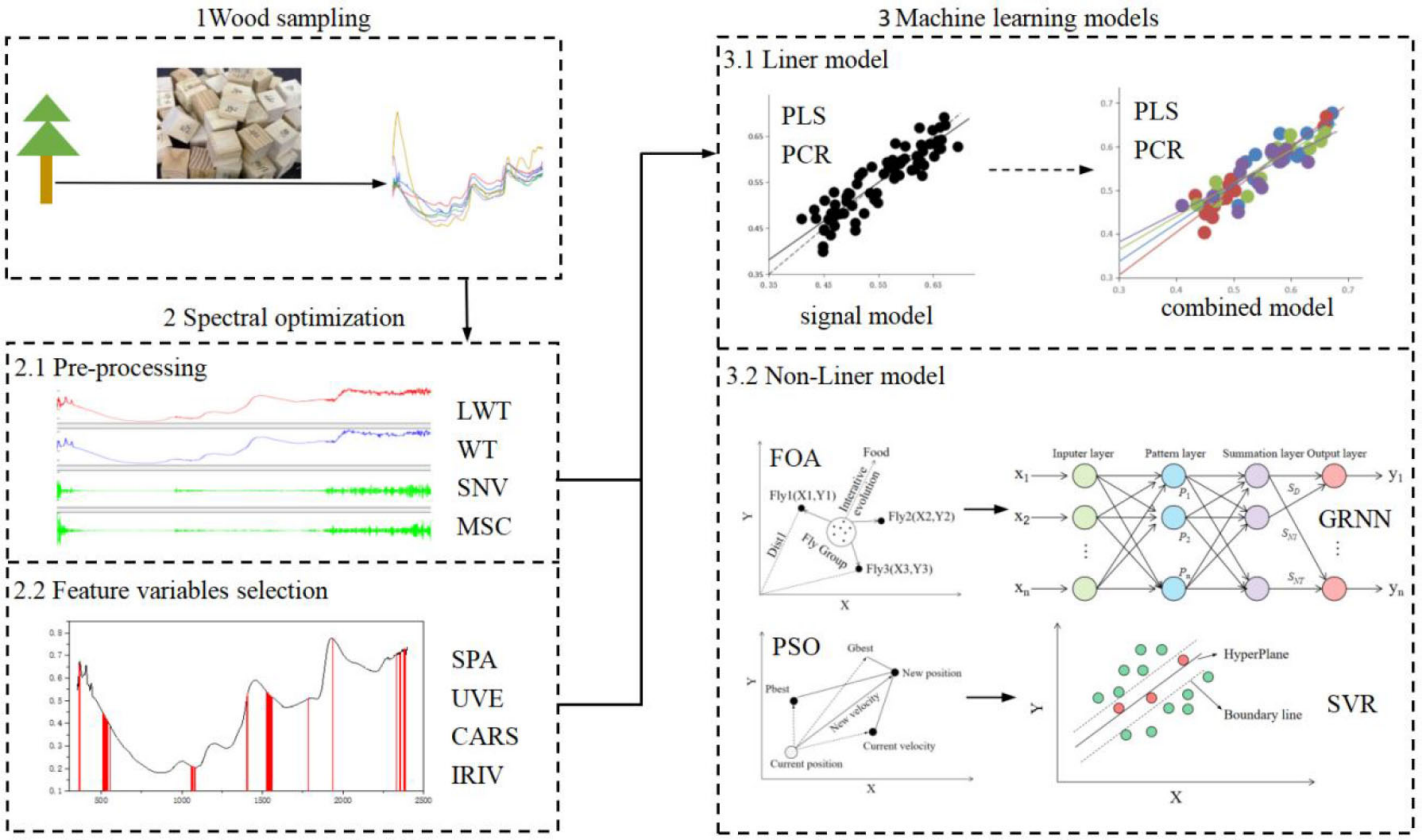
The respective work flow of chemometric methods.

Linear modeling method, namely PLS model, was used for determining the optimal pre-processing and variable selection method. Additionally, non-linear deep learning techniques including GRNN and PSO-SVM were performed using Matlab based on the selected variables. The parameter of Spread is of great importance for GRNN model. As FOA was applied to select the optimal Spread value. As for PSO-SVM model, PSO was used to optimize the parameters of penalty factor (C) and kernel function (g) in the radial basis function (RBF) kernel. Furthermore, in order to improve the accuracy of PSO-SVM model, three parameters of PSO-SVM, namely cross-validation number, maximum generation, and population, were optimized by Box-Behnken design of RSM method. GRNN and PSO-SVM models were established in Matlab. RSM was performed in Design-Expert Software 11 (Stat-Ease, Minneapolis, Minnesota, USA) (**Figure 2**).

## 3 Results

### 3.1 Wood density analysis

**Figure 3:**
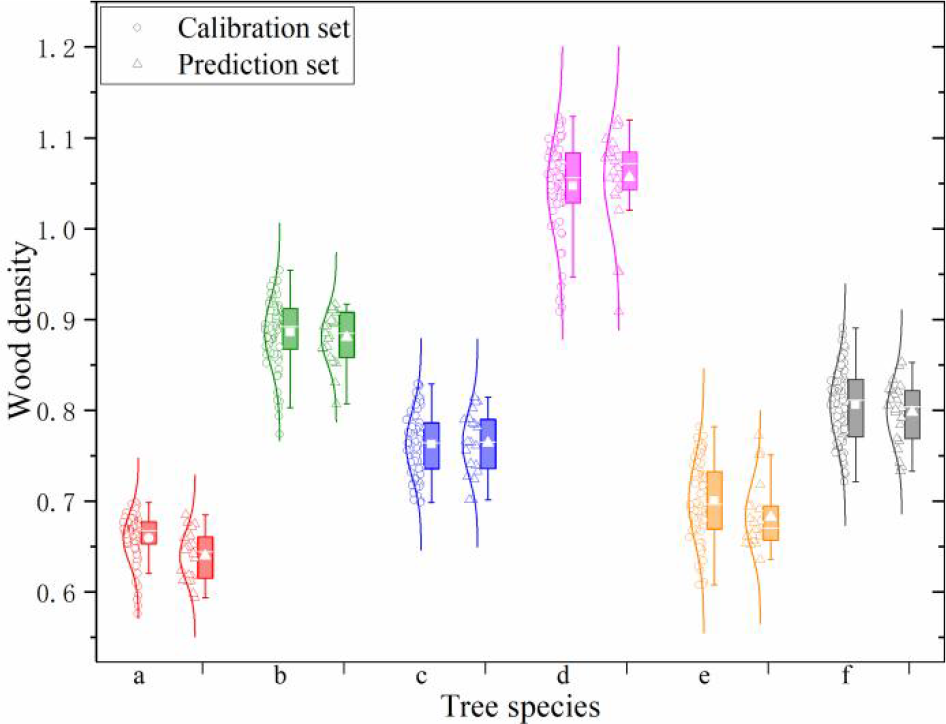
Statistical descriptive of wood density. (a–f) are Tilia tuan Szyszyl., Acer mono Maxim, Chinese white poplar (Jilin), Japanese elm, Chinese white poplar (Heilongjiang), and Dahurian larch, respectively.

For evaluation the performance of various chemometric methods on different tree species, the calibration set and prediction set for each tree species were divided using random sampling method. The statistical descriptive of wood density is demonstrated in **Figure 3**. Wood density values ranged from 0.576 to 1.124 g/cm^3^ among these tree species. The mean density value was different. Japanese elm from Jilin and Heilongjiang province presented a large mean value (1.047 and 1.058 g/cm^3^, respectively) and a higher standard deviation. In terms of data set, the range of density in the prediction set was within the corresponding calibration set. Additionally, regardless of the type of tree species, a similar mean value and standard deviation were obtained for a determined tree species between calibration and prediction set.

### 3.2 Comparison of various spectral de-noising methods

The selection of wavelet function is the first step of de-noising based on LWT or WT. **Table 1** depicts the PLS models accuracy of four wavelet functions when the wavelet order and decomposition level were assumed to be 3 and 8, respectively. The overall accuracy of calibration set is higher than 0.7, regardless of the wavelet function used. For the precision of a determined tree species, various performance were obtained among these four wavelet functions. For example, different values of cross-validation set were achieved for Tilia tuan Szyszyl. when sym3 and bior1.3 functions were used. In contrast, db3 performs good with respect to the high R^2^, RPD value and lower RMSE, RSD value for Tilia tuan Szyszyl., Acer mono Maxim, Japanese elm, and Dahurian larch. However, the same tree species of Chinese white poplar from Jilin and Heilongjiang province are not identical, with the optimal wavelet function of sym3 and bior1.3, respectively. In addition, the parameters of PLS models are different among these two locations. These demonstrated that geographical origin has impact on the prediction of density and spectral pre-processing based on Vis-NIR spectroscopy and LWT.

**Table 1:** The PLS model results of wood density with different wavelet functions.

Location	Tree species	Models	Indicators	Wavelet function			
				Haar	db3	sym3	bior1.3
Jilin province	*Tilia tuan* Szyszyl.	Calibration	R^2^	0.812	0.823	0.814	0.814
			RMSE	0.012	0.011	0.012	0.012
			RPD	2.306	2.377	2.319	2.319
			RSD	1.820%	1.668%	1.820%	1.820%
		Cross-validation	R^2^	0.800	0.801	0.803	0.812
			RMSE	0.012	0.012	0.012	0.012
			RPD	2.236	2.242	2.253	2.306
			RSD	1.820%	1.820%	1.820%	1.820%
	Acer mono Maxim	Calibration	R^2^	0.755	0.740	0.759	0.748
			RMSE	0.018	0.019	0.018	0.018
			RPD	2.02	1.961	2.037	1.992
			RSD	2.031%	2.144%	2.031%	2.031%
		Cross-validation	R^2^	0.639	0.654	0.705	0.665
			RMSE	0.022	0.022	0.021	0.021
			RPD	1.664	1.7	1.841	1.728
			RSD	2.483%	2.483%	2.370%	2.370%
	Chinese white poplar	Calibration	R^2^	0.763	0.780	0.761	0.769
			RMSE	0.017	0.017	0.017	0.017
			RPD	2.054	2.132	2.046	2.081
			RSD	2.228%	2.228%	2.228%	2.228%
		Cross-validation	R^2^	0.652	0.650	0.679	0.657
			RMSE	0.021	0.021	0.020	0.021
			RPD	1.695	1.69	1.765	1.707
			RSD	2.753%	2.753%	2.622%	2.753%
	Japanese elm	Calibration	R^2^	0.843	0.857	0.854	0.825
			RMSE	0.020	0.020	0.020	0.022
			RPD	2.524	2.644	2.617	2.39
			RSD	1.910%	1.910%	1.910%	2.101%
		Cross-validation	R^2^	0.826	0.829	0.823	0.807
			RMSE	0.022	0.022	0.022	0.023
			RPD	2.397	2.418	2.377	2.276
			RSD	2.101%	2.101%	2.101%	2.196%
Heilongjiang province	Chinese white poplar	Calibration	R^2^	0.760	0.749	0.749	0.758
			RMSE	0.022	0.022	0.022	0.022
			RPD	2.041	1.996	1.996	2.033
			RSD	3.140%	3.140%	3.140%	3.140%
		Cross-validation	R^2^	0.705	0.689	0.726	0.730
			RMSE	0.024	0.025	0.024	0.024
			RPD	1.841	1.793	1.91	1.925
			RSD	3.425%	3.568%	3.425%	3.425%
	Dahurian larch	Calibration	R^2^	0.720	0.734	0.735	0.716
			RMSE	0.021	0.021	0.021	0.022
			RPD	1.89	1.939	1.943	1.876
			RSD	2.605%	2.605%	2.605%	2.729%
		Cross-validation	R^2^	0.684	0.684	0.653	0.659
			RMSE	0.023	0.023	0.024	0.024
			RPD	1.779	1.779	1.698	1.712
			RSD	2.853%	2.853%	2.977%	2.977%

**Figure 4:**
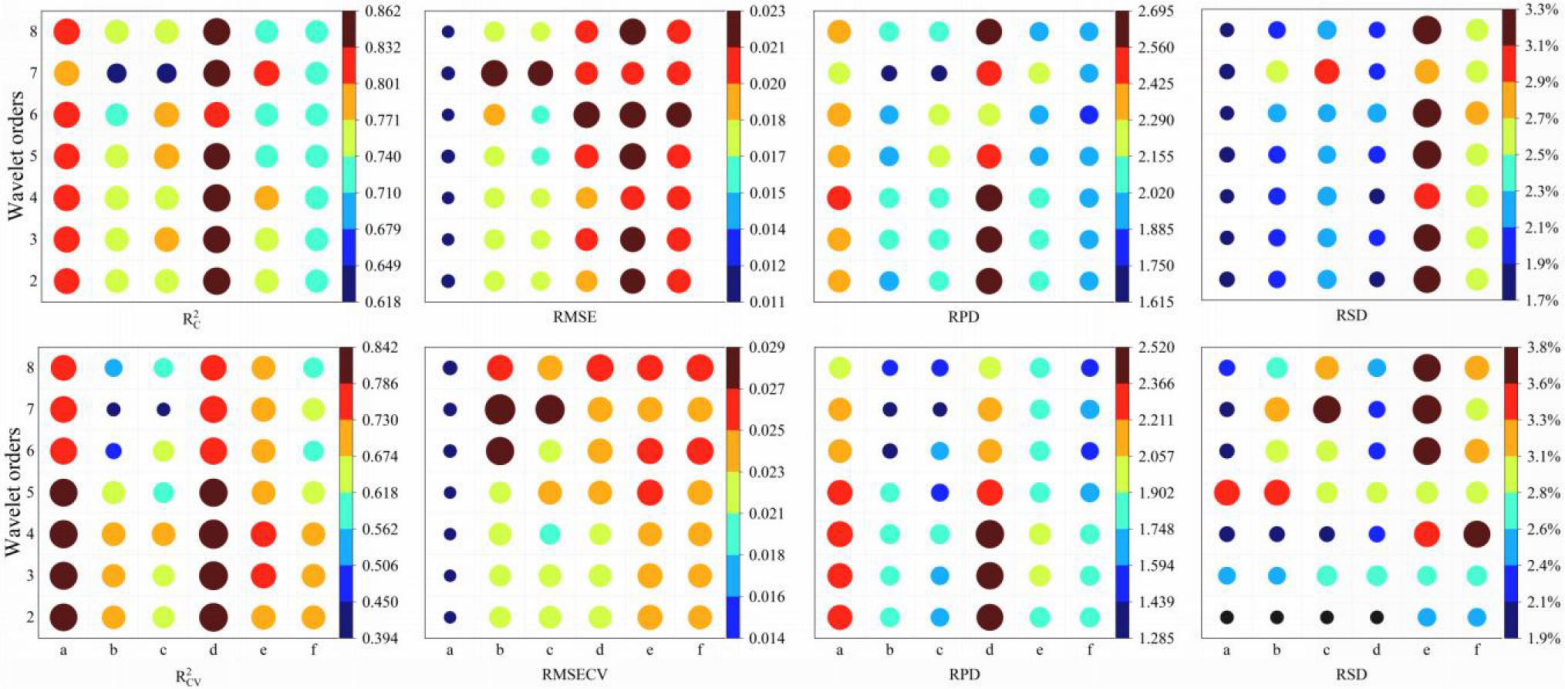
Results of PLS models for wood density with different wavelet orders. (a–f) are *Tilia tuan Szyszyl*., Acer mono Maxim, Chinese white poplar (Jilin), Japanese elm, Chinese white poplar (Heilongjiang), and Dahurian larch, respectively.

**Figure 4** displays the variation of PLS models using the optimal wavelet function in relation to the increasing wavelet orders from 2 to 8. The results show that a non-obvious trend was received with the enhance of wavelet orders. This is consistent with the results of Zhang et al. (Zhang et al., 2009). In contrast, the performance is relatively good for *Tilia tuan* Szyszyl., Acer mono Maxim, Japanese elm, and Dahurian larch when the wavelet order equals 4. In terms of Chinese white poplar from Jilin and Heilongjiang province, similar to the results of wavelet function, the optimal wavelet order is different with the values of 5 and 6, respectively. Additionally, for *Tilia tuan* Szyszyl., Acer mono Maxim, and Dahurian larch, the RMSE values of calibration and cross-validation set are similar, but the R^2^ and RPD values slightly outperform the other orders when the order is 4.

**Figure 5:**
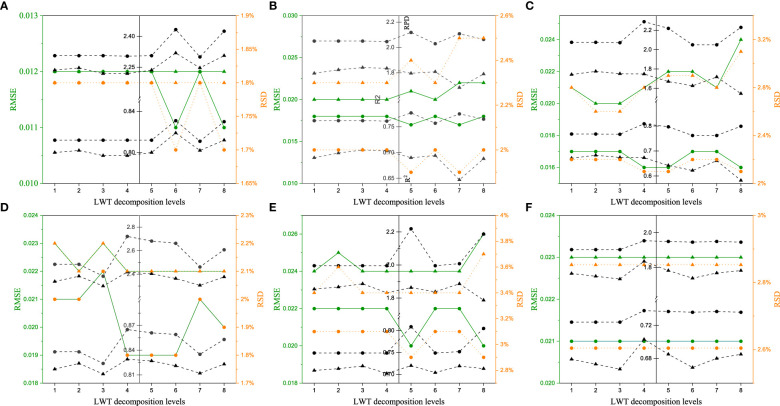
Results of PLS models for wood density with different decomposition levels. **(A–F)** are *Tilia tuan Szyszyl*., Acer mono Maxim, Chinese white poplar (Jilin), Japanese elm, Chinese white poplar (Heilongjiang), and Dahurian larch, respectively.

When the optimal wavelet function and order were determined for these tree species, the performance of PLS models using various decomposition level (1-8) was analyzed. Unlike the results of wavelet order, **Figure 5** illustrates that despite the similar RMSE values for a determined tree among various decomposition levels, the R^2^ and RPD reach the largest value when the decomposition level increases from 1 to 8. This can be explained that the noise were removed from spectra with the increasing of decomposition level, while the useful information were regarded as noise when the decomposition level is too large. Considering the performance of calibration set and cross-validation set, the optimal decomposition level is 4 for Chinese white poplar (Jilin province), Japanese elm, and Dahurian larch. Acer mono Maxim and Chinese white poplar performed well when the decomposition level equals 5. For *Tilia tuan* Szyszyl., the optimal decomposition level is 6. As for Chinese white poplar harvested from two locations, the results of the optimal decomposition levels were similar to wavelet orders.

**Figure 6:**
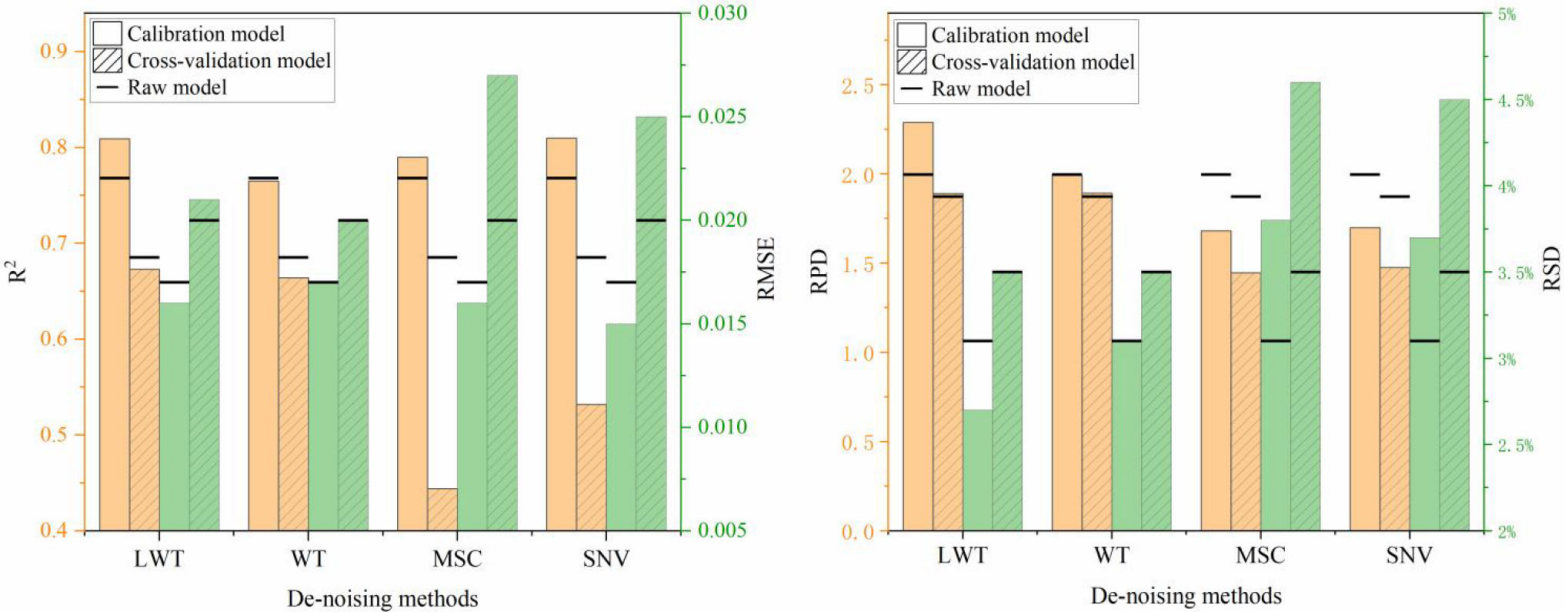
The comparison analysis of various de-noising methods for Chinese white poplar (Heilongjiang).

Considering the feasibility of LWT, the performance of the three traditional pre-processing techniques was further compared. Only the data of Chinese white poplar from Heilongjiang was shown in **Figure 6**. The results show that the LWT performs better with respect to the R^2^ and RPD values in the calibration and cross-validation dataset. The RMSE and RSD values were smaller than that of corresponding raw model. In contrast, the performance of WT, MSC, and SNV was worse than LWT with a lower R^2^ and RPD of cross-validation model, especially for the latter two methods. These results demonstrated that the PLS models using MSC and SNV approaches involve the overfitting problem in the prediction of wood density.

### 3.3 Feature variable selection of wood density

**Figure 7:**
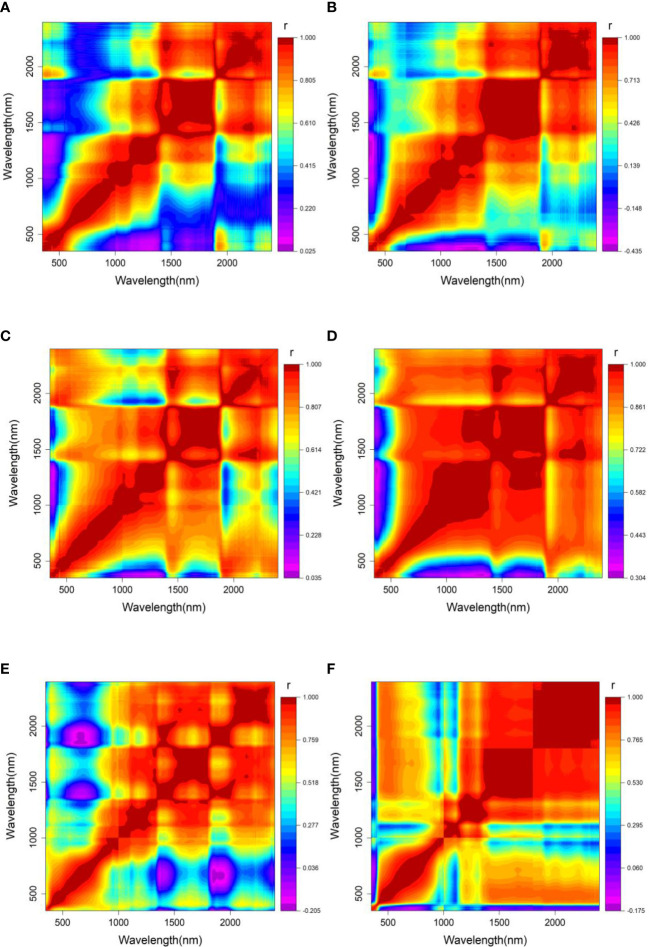
2-D correlation spectra of wavelength variables for each tree species with log(1/R) spectra. **(A–F)** are *Tilia tuan* Szyszyl., Acer mono Maxim, Chinese white poplar (Jilin), Japanese elm, Chinese white poplar (Heilongjiang), and Dahurian larch.

**Figure 7** illustrates two dimensional (2D) correlation spectroscopy between wavelengths of various wood spectra. Regardless of tree species, the high correlation values (r) indicate that more redundant information or collinearity were exhibited, especially for Chinese white poplar (Jilin) (**Figure 7C**), Japanese elm (**Figure 7D**), and Dahurian larch (**Figure 7F**). Additionally, the correlation of adjacent spectral variables was higher than other regions, which increases the computation time and complexity in modeling. Therefore, four variable selection approaches, SPA, UVE, CARS, and IRIV, were employed to select the feature variable related to wood density and decrease the redundant information.

**Table 2** displays the prediction accuracy of four variable selection approaches on wood density using PLS models. For the number of selected variables, different numbers were obtained for UVE, CARS, and IRIV among these tree species, except for SPA. When using the SPA, 60 wavelengths were selected for Tilia tuan Szyszyl. and Chinese white poplar (Jilin), Chinese white poplar (Heilongjiang) and Dahurian larch also have the same numbers of selected variables (64). However, the indicators of PLS models were different, for example, the R^2^ values of calibration set are 0.839 and 0.775 for Tilia tuan Szyszyl. and Chinese white poplar (Jilin), respectively. In terms of precision, the performance of cross-validation set was worse than that of calibration set for SPA. This demonstrated that despite a dimensionality reduction in the spectral matrix, SPA method has a overfitting problem for the density prediction among these tree species. In contrast, CARS and IRIV have stable and better results for calibration and validation set. The optimal variable selection method of Tilia tuan Szyszyl. and Chinese white poplar (Jilin and Heilongjiang) is CARS, and IRIV performed better than other methods for Acer mono Maxim, Japanese elm, and Dahurian larch.

**Table 2:** The comparison of various variable selection methods for each tree species.

Tree species	No. of Variables	${\text{R}}_{\text{c}}^{2}$	RMSE	RSD	RPD	No. of Variables	${\text{R}}_{\text{c}}^{2}$	RMSE	RSD	RPD
Full-PLS						LWT-PLS				
*Tilia tuan* Szyszyl.	2048	0.812	0.012	1.820%	2.306	2048	0.831	0.011	1.668%	2.433
*Acer mono Maxim*	2048	0.762	0.018	2.031%	2.05	2048	0.777	0.017	1.918%	2.118
*Chinese white poplar*	2048	0.768	0.017	2.228%	2.076	2048	0.809	0.016	2.097%	2.288
*Japanese elm*	2048	0.838	0.021	2.005%	2.485	2048	0.865	0.019	1.184%	2.722
*Chinese white poplar*	2048	0.749	0.022	3.140%	1.996	2048	0.809	0.019	2.711%	2.288
*Dahurian larch*	2048	0.724	0.021	2.605%	1.903	2048	0.738	0.021	2.605%	1.954
SPA-PLS						UVE-PLS				
*Tilia tuan* Szyszyl.	60	0.839	0.011	1.668%	2.492	1249	0.825	0.011	1.668%	2.39
*Acer mono Maxim*	69	0.782	0.018	2.301%	2.142	800	0.745	0.018	2.031%	1.98
*Chinese white poplar*	60	0.775	0.017	2.228%	2.108	733	0.679	0.020	2.622%	1.765
*Japanese elm*	73	0.840	0.021	2.005%	2.5	1139	0.841	0.021	2.005%	2.508
*Chinese white poplar*	64	0.765	0.022	3.140%	2.063	410	0.679	0.026	3.710%	1.765
*Dahurian larch*	64	0.682	0.023	2.853%	1.773	418	0.729	0.021	2.605%	1.921
CARS-PLS						IRIV-PLS				
*Tilia tuan* Szyszyl.	992	0.828	0.011	1.668%	2.411	5	0.806	0.012	1.820%	2.27
*Acer mono Maxim*	62	0.777	0.017	1.918%	2.118	20	0.819	0.016	1.805%	2.351
*Chinese white poplar*	35	0.800	0.016	2.097%	2.236	10	0.779	0.021	2.753%	2.127
*Japanese elm*	47	0.874	0.018	1.719%	2.817	25	0.901	0.012	1.146%	3.178
*Chinese white poplar*	23	0.783	0.021	2.997%	2.147	16	0.755	0.022	3.140%	2.02
*Dahurian larch*	13	0.767	0.020	2.481%	2.072	15	0.800	0.018	2.232%	2.236

**Figure 8:**
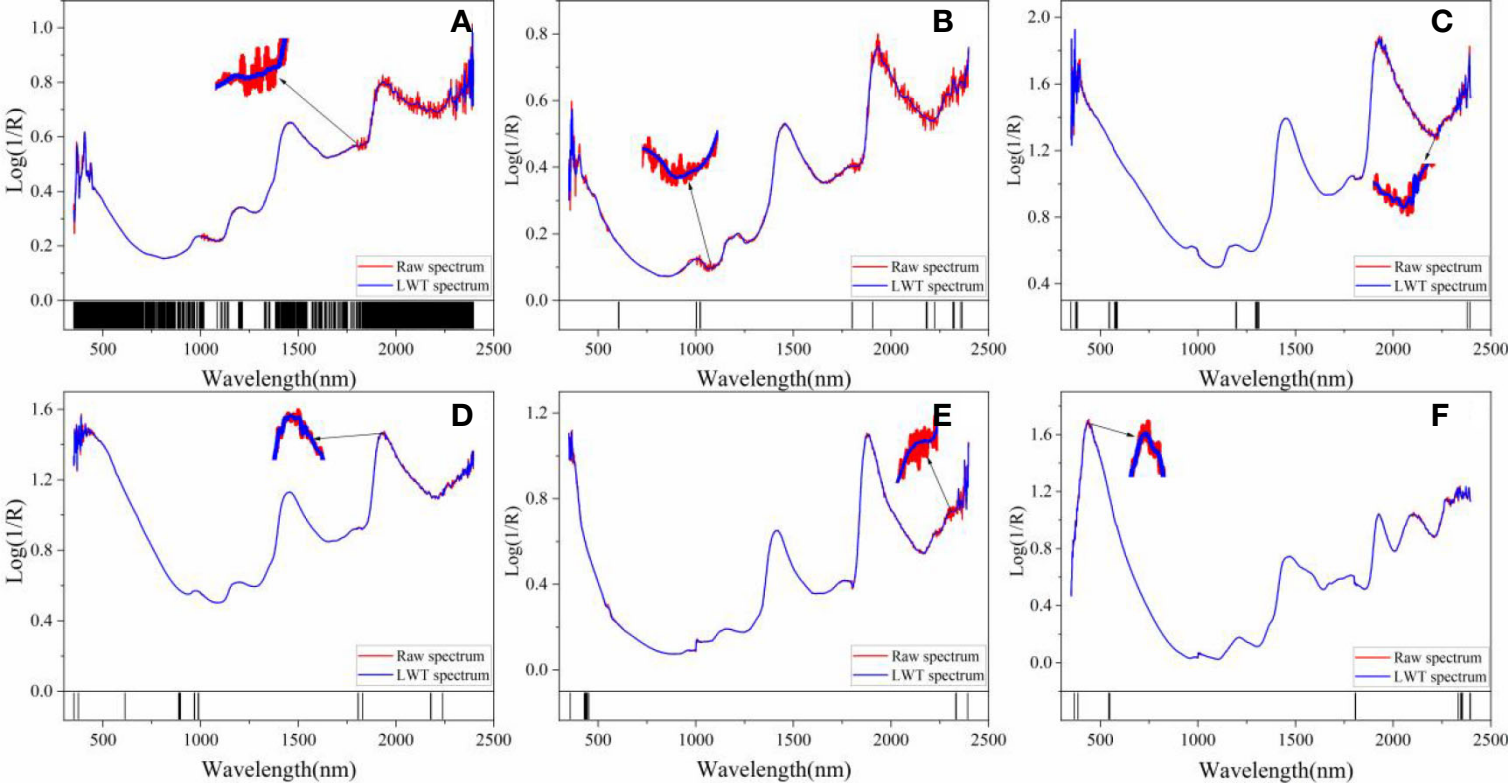
The distributions of selected variables by the optimal method for each tree species. **(A–F)** are *Tilia tuan* Szyszyl., Acer mono Maxim, Chinese white poplar (Jilin), Japanese elm, Chinese white poplar (Heilongjiang), and Dahurian larch, respectively.

The distributions of selected variables for these tree species using the optimal method are shown in **Figure 8**. The selected bands of CARS and IRIV, 1157, 1171, 1370, 1597, 1811, 1830, 2200, and 2353 nm, are associated with hemicellulose, cellulose, and lignin (Ali et al., 2001; Sandak et al., 2011; Schwanninger et al., 2011; Yonenobu and Tsuchikawa, 2003). This results are consistent with our previous studies (Li et al., 2020), indicating that the determination of wood density are related to chemical compounds. Additionally, comparing of the raw spectra and de-noising spectra with LWT for these tree species (**Figure 8**), it can be found that the LWT makes the wood spectra smooth and has a similar trend with the corresponding raw spectra.

#### 3.3.1 The optimization of non-linear calibration models

**Figure 9:**
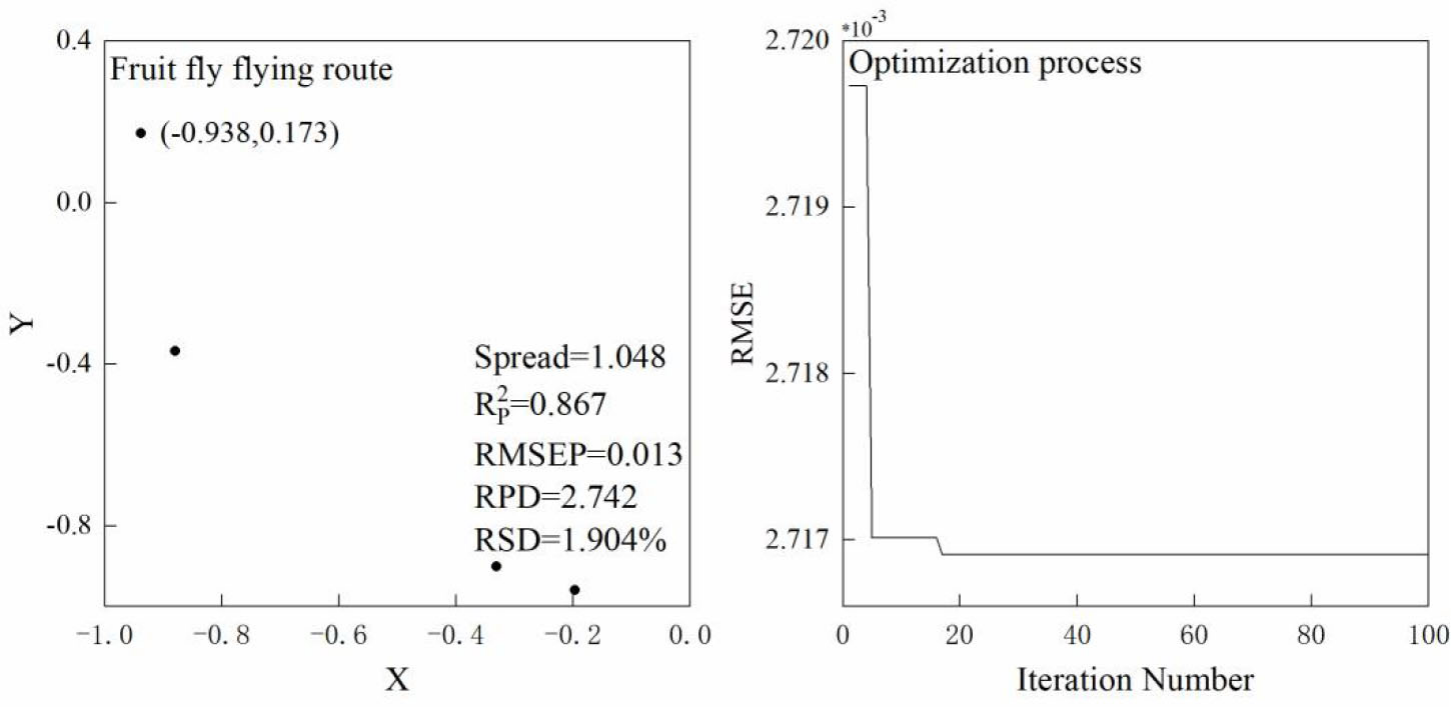
The optimization process of FOA-GRNN models for Chinese white poplar (Heilongjiang).

When the wood spectra were pre-processed by the optimal de-noising and variable selection methods, GRNN and SVM were used to build non-linear models based on these selected variables. In addition, FOA and PSO were employed to optimize the parameter of GRNN (Spread value) and SVM (penalty factor and kernel function), respectively. Regardless of tree species, the prediction accuracy resulting from LWT dataset was higher than that of raw prediction set. Considering four indicators, Chinese white poplar (Heilongjiang) delivers the best performance based on LWT dataset with R^2^, RMSE, RPD, and RSD value of 0.867, 0.013, 2.742 and 1.904%, respectively (**Figure 9**). Compared to FOA-GRNN models, four indicators are better, regardless of the pretreatment of prediction set, when using the P SO-SVM, apart from Japanese elm and Chinese white poplar (Heilongjiang) (**Table 3**). These results show that tree species have affect on the selection of the optimal chemometric method.

**Table 3:** The results of PSO-SVM models for prediction sets with different pretreatment.

Dataset	Location	Tree species	Variables	Calibration model				Prediction model			
				R^2^	RMSE	RPD	RSD	R^2^	RMSE	RPD	RSD
Prediction set with raw spectra	Jilin province	*Tilia tuan* Szyszyl.	992	0.908	0.008	3.297	1.213%	0.785	0.012	2.157	1.875%
		Acer mono Maxim	20	0.815	0.016	2.325	1.805%	0.725	0.015	1.907	1.703%
		Chinese white poplar	35	0.767	0.017	2.072	2.228%	0.721	0.018	1.893	2.355%
		Japanese elm	250	0.876	0.018	2.840	1.719%	0.815	0.022	2.325	2.082%
	Heilongjiang province	Chinese white poplar	23	0.784	0.021	2.152	2.997%	0.738	0.018	1.954	2.637%
		Dahurian larch	15	0.802	0.018	2.247	2.232%	0.733	0.017	1.935	2.130%
Prediction set with LWT de-noising	Jilin province	*Tilia tuan* Szyszyl.	992	0.915	0.008	3.430	1.213%	0.787	0.012	2.167	1.875%
		Acer mono Maxim	20	0.817	0.016	2.338	1.805%	0.727	0.015	1.914	1.703%
		Chinese white poplar	35	0.773	0.017	2.099	2.228%	0.734	0.018	1.939	2.355%
		Japanese elm	250	0.889	0.017	3.002	1.623%	0.830	0.021	2.425	1.987%
	Heilongjiang province	Chinese white poplar	23	0.784	0.021	2.152	2.997%	0.746	0.018	1.984	2.637%
		Dahurian larch	15	0.811	0.018	2.300	2.232%	0.747	0.017	1.988	2.130%

**Figure 10:**
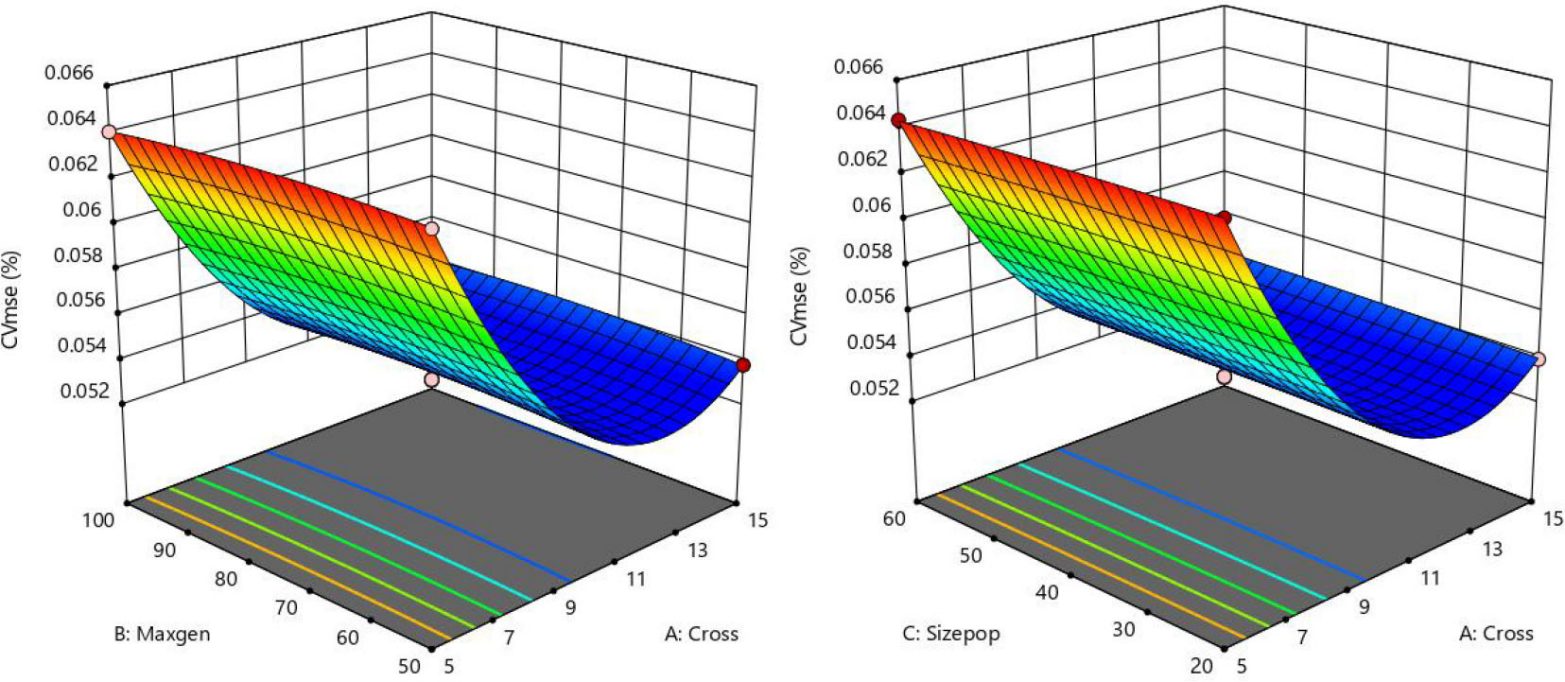
Response surface plot for interactions between three variables on CVmse for PSO-SVM models of Dahurian larch.

As for PSO-SVM model, although the PSO was used to optimize the parameters of penalty factor and kernel function, three parameters, including population size, maximum generation, and the No. of cross-validation, also have influence on the performance of modeling. This may be the reason of low accuracy of Japanese elm and Chinese white poplar (Heilongjiang). Therefore, the Box-Behnken design with a three-factor and three-level of RSM was used to analyze the relationship between these three parameters and model performance. According to the experimental values and coded levels of these three factors (**Table 4**), the relationship between three parameters and mean squared error of cross-validation (CVmse) for Dahurian larch are shown in **Figure 10**.

**Table 4:** Experimental values and coded levels of variable using Box–Behnken design.

Factor Levels	Variable		
	No. of cross-validation (A)	Maximum generation (B)	Population size (C)
-1	5	50	20
0	10	75	40
1	15	100	60

**Figure 10** displays the response surface plot between three parameters and CVmse. The optimal parameter was determined when the CVmse has the lowest value. In terms of Dahurian larch, the CVmse value first reduced and then increased with the cross-validation number increasing from 5 to 15, when the maximum generation is a certain value. The lowest value of CVmse was achieved with the No. of cross-validation, maximum generation, and population size at 10, 75, and 40, respectively. According to the ANOVA results of Dahurian larch (data not shown), the CVmse was significantly influenced by cross-validation number (p<0.01) than by population size and maximum generation. For *Tilia tuan* Szyszyl. (data not shown), the minimum CVmse was obtained when the No. of cross-validation, maximum generation, and population size were 5, 50, and 20, respectively.

**Figure 11:**
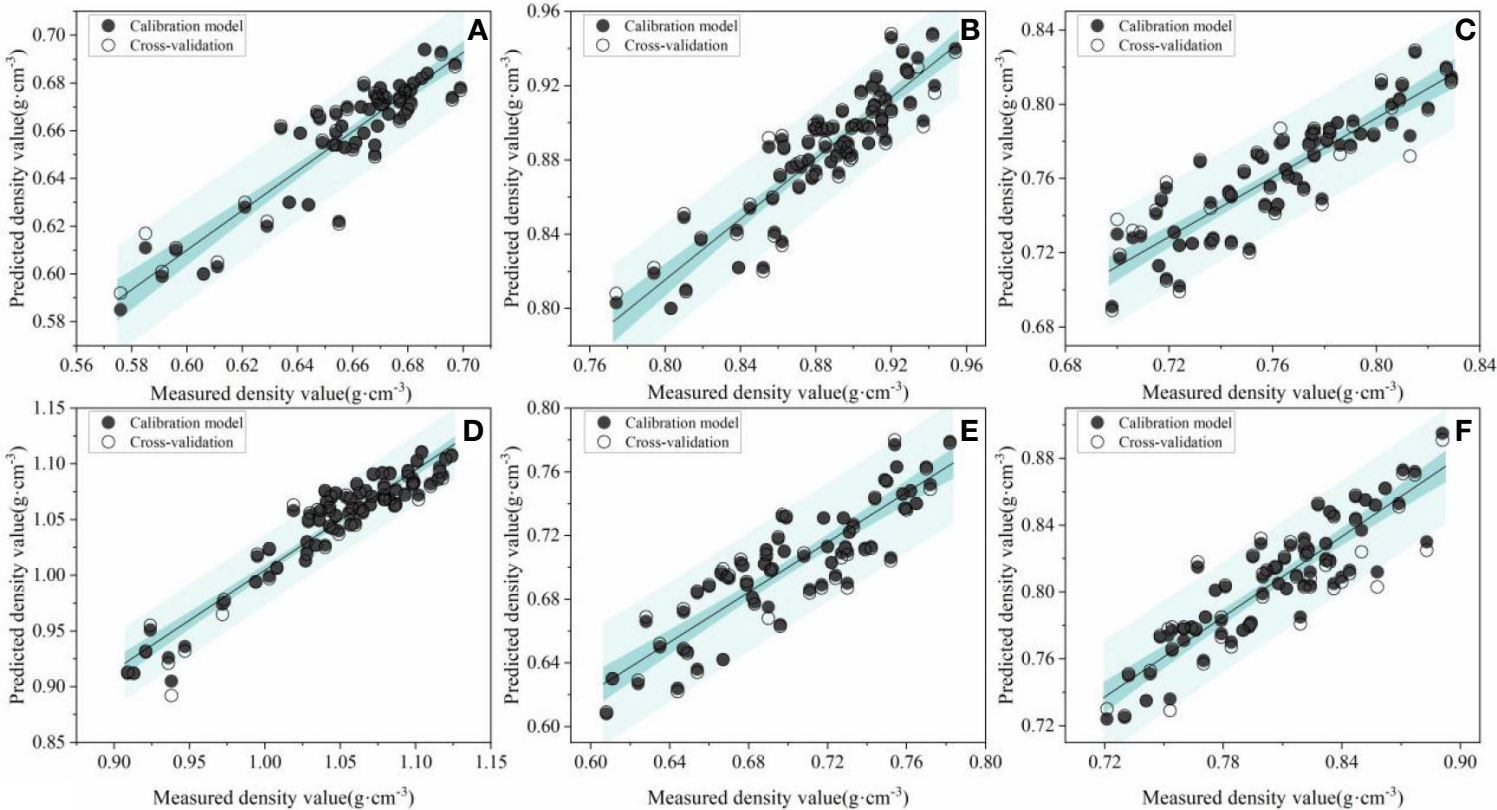
The scatter plots of predicted and measured values for RSM-PSO-SVM models. **(A–F)** are *Tilia tuan* Szyszyl., Acer mono Maxim, Chinese white poplar (Jilin), Japanese elm, Chinese white poplar (Heilongjiang), and Dahurian larch, respectively.

**Figure 11** shows the wood density accuracy of RSM-PSO-SVM models based on the Box-Behnken design. In terms of accuracy, Japanese elm delivers the best performance with the R^2^ of 0.955 and 0.862 for calibration and prediction set, respectively. For Acer mono Maxim, the improvement of $R_{p}^{2}$ value slightly outperforms corresponding ${\text{R}}_{\text{c}}^{2}$ value. This indicates that RSM-PSO-SVM models are more stable than that of PSO-SVM models. However, RSM-PSO-SVM had a poor performance for Chinese white poplar (Heilongjiang) ( ${\text{R}}_{\text{p}}^{2}$ =0.752, RMSEP=0.018, RPD=2.008, RSD=2.637%) when comparing with FOA-GRNN model ( ${\text{R}}_{\text{p}}^{2}$ =0.867,RMSEP=0.013, RPD=2.742, RSD=1.904%). These results demonstrated that there is not a universal chemometric method that works for all scenarios.

## 4 Discussions

Researchers pay more attention to spectral de-noising or feature variable selection to reduce the influence of irrelevant or interference information in the spectral analysis. A comparison of various chemometric methods including spectral pre-processing, feature variable selection, and the optimization of non-linear calibration models in the wood density prediction among different tree species and geographical origins simultaneously were first analyzed using Vis-NIR spectroscopy in this study. The results demonstrated that LWT outperform WT, MSC, SNV, and raw spectra among these tree species for wood spectra optimization. There are few studies on LWT and WT de-noising in NIR spectra analysis. Abasi et al. (2019) employed WT to optimize Gala apple Vis-NIR spectra in the determination of quality parameters. The R^2^ values were higher than 0.85 for soluble solids content, moisture content, and pH. In the forestry field, WT (Daubechies-5, db5) and LWT (db2) were used to optimize Populus davidiana and larch spectra, respectively in our previous studies (Li et al., 2018a; Li et al., 2018b). In this study, LWT with four wavelet functions including Haar, sym3, db3, and bior1.3 were compared simultaneously among various tree species from two locations. The results demonstrated that there has differences in the optimal de-noising parameters of LWT among these tree species. Therefore, an appropriate pre-processing technique should be selected before building models.

As for feature variable selection. compared to full spectra de-noised by LWT, CARS and IRIV achieved the best results, and the spectral dimensionality was reduced by 51.56%, 99.02%, 98.29%, 98.78%, 98.88%, and 99.27% for Tilia tuan Szyszyl., Acer mono Maxim, Chinese white poplar (Jilin), Japanese elm, Chinese white poplar (Heilongjiang), and Dahurian larch, respectively. In terms of non-linear models, RSM-PSO-SVM delivers the best performance than corresponding FOA-GRNN models, except for Chinese white poplar harvest from Heilongjiang province. In order to better analyze the performance of these chemometric methods, two traditional linear models, i.e., PLS and PCR, were employed to build signal and combined models using raw spectra, respectively. Signal model is a model that includes one tree species. And combined model is a model that includes all tree species and geographical locations simultaneously. The results of these two kinds of models are shown in **Table 5**, respectively.

**Table 5:** The results of PLS and PCR signal and combined models for various tree species.

Models	Indicators	Signal models						Combined models
		Jilin province			Heilongjiang province			/
		*Tilia tuan* Szyszyl.	Acer mono Maxim	Chinese white poplar	Japanese elm	Chinese white poplar	Dahurian larch	/
PLS	$R_{\text{c}}^{2}$	0.812	0.762	0.768	0.838	0.749	0.724	0.660
	RMSEC	0.012	0.018	0.017	0.021	0.022	0.021	0.080
	RPD	2.306	2.05	2.076	2.485	1.996	1.903	1.715
	RSD	1.820%	2.031%	2.228%	2.005%	3.140%	2.605%	9.771%
	$R_{\text{cv}}^{2}$	0.799	0.722	0.685	0.825	0.715	0.690	0.561
	RMSECV	0.012	0.020	0.020	0.022	0.024	0.023	0.091
	RPD	2.23	1.897	1.782	2.39	1.873	1.796	1.509
	RSD	1.820%	2.257%	2.622%	2.101%	3.425%	2.853%	11.115%
PCR	$R_{\text{c}}^{2}$	0.785	0.751	0.752	0.836	0.725	0.703	0.546
	RMSEC	0.013	0.018	0.018	0.021	0.023	0.022	0.092
	RPD	2.157	2.004	2.008	2.469	1.907	1.835	1.484
	RSD	1.972%	2.031%	2.360%	2.005%	3.282%	2.729%	11.237%
	$R_{\text{cv}}^{2}$	0.770	0.712	0.668	0.813	0.699	0.685	0.507
	RMSECV	0.013	0.020	0.021	0.022	0.024	0.023	0.097
	RPD	2.085	1.863	1.736	2.312	1.823	1.782	1.424
	RSD	1.972%	2.257%	2.753%	2.101%	3.425%	2.853%	11.847%

Comparison of the accuracy of PLS and PCR method based on raw spectra (**Table 5**), the calibration set outperforms the cross-validation set with a higher R^2^, RPD and smaller RMSE, RSD value, regardless of signal and combined models. In addition, PLS model provides high accuracy on various wood density prediction than that of PCR model. However, the performance of combined models was worse than signal model, especially for PCR method.

**Table 6**, **7** show the prediction accuracy of signal models and combined models, respectively. Similar to the results of calibration set, the PLS method achieved a higher prediction accuracy than PCR model. Additionally, the combined model delivers the worst performance on prediction dataset. Compared to the optimal model of RSM-PSO-SVM, the PLS approach is relatively bad except the Chinese white poplar from Jilin province.

**Table 6:** The prediction results of signal models for each tree species.

Models	Indicators	Jilin province				Heilongjiang province	
		*Tilia tuan* Szyszyl.	Acer mono Maxim	Chinese white poplar	Japanese elm	Chinese white poplar	Dahurian larch
PLS	$R_{\text{p}}^{2}$	0.771	0.522	0.758	0.855	0.797	0.714
	RMSEP	0.107	0.144	0.110	0.076	0.096	0.114
	RPD	2.090	1.446	2.033	2.626	2.219	1.870
	RSD	16.722%	16.352%	14.395%	7.191%	14.062%	14.282%
PCR	$R_{\text{p}}^{2}$	0.648	0.520	0.736	0.838	0.714	0.644
	RMSEP	0.133	0.144	0.115	0.081	0.114	0.127
	RPD	1.685	1.443	1.946	2.485	1.870	1.676
	RSD	20.785%	16.352%	15.049%	7.664%	16.699%	15.911%

**Table 7:** The prediction results of combined models for each tree species.

Location	Tree species	Indicators	Combined models with PLS				Combined models with PCR			
			All tree species	Jilin	Heilong jiang	Chinese white poplar	All tree species	Jilin	Heilong jiang	Chinese white poplar
Jilin province	*Tilia tuan* Szyszyl.	$R_{\text{p}}^{2}$	–	–			–	–		
		RMSEP	–	–			–	–		
		RPD	–							
		RSD	–							
	Acer mono Maxim	$R_{\text{p}}^{2}$	–	–			–	–		
		RMSEP	–	–			-	–		
		RPD	–							
		RSD	–							
	Chinese white poplar	$R_{\text{p}}^{2}$	–	–		0.496	–	–		0.445
		RMSEP	–	–		0.159	–	–		0.167
		RPD	–			1.409				1.342
		RSD	–			20.807%				21.854%
	Japanese elm	$R_{\text{p}}^{2}$	–	–			–	–		
		RMSEP	–	–			–	–		
		RPD	–							
		RSD	–							
Heilongjiang province	Chinese white poplar	$R_{\text{p}}^{2}$	–		0.402	0.714	–		0.386	0.463
		RMSEP	–		0.165	0.114	–		0.167	0.156
		RPD	–		1.293	1.870			1.276	1.365
		RSD	–		24.170%	16.699%			24.463%	22.851%
	Dahurian larch	$R_{\text{p}}^{2}$	–		–		–		–	
		RMSEP	–		–		–		–	
		RPD	–							
		RSD	–							

Blank indicates that no experiment is scheduled, “-” indicates that the prediction result is negative.

## 5 Conclusions

This study demonstrates the feasibility of using Vis-NIR spectra combined with various chemometric methods including spectral de-noising, feature variables selection, and the optimization of modeling parameters, to predict wood density. LWT is excellent for spectral de-noising when comparing traditional methods. CARS and IRIV outperforms in the feature variables selection among these tree species. In terms of linear and non-linear calibration models, RSM-PSO-SVM delivers the best performance except for Chinese white poplar. The optimal model of Chinese white poplar from Jilin and Heilongjiang province are PLS and FOA-GRNN model, respectively. These results indicate that the geographical location has an effect on the selection of chemometric methods in wood density determination. Additionally, in order to overcome the difference of the optimal model, model transfer will be used to predict wood properties of different locations in future studies.

## Data availability statement

The raw data supporting the conclusions of this article will be made available by the authors, without undue reservation.

## Author contributions

YiL analyzed the data and wrote the original draft. BV contributed to editing. FH performed formal analysis. ZP performed supervision. All authors contributed to the article and approved the submitted version.
